# Supplementary material for: Interferon Alpha Therapy Increases Pro-Thrombotic Biomarkers in Patients with Myeloproliferative Neoplasms
Source: Cancers (Basel). 2020 Apr 17;12(4):992. doi: 10.3390/cancers12040992 (PMC7226618; doi:10.3390/cancers12040992)
Supplement: Supplementary file 1 [file cancers-12-00992-s001.pdf]

Supplementary Materials

Interferon Alpha Therapy Increases Pro-Thrombotic Biomarkers in Patients with Myeloproliferative Neoplasms

Dorothée Faille, Lamia Lamrani, Stéphane Loyau, Marie-Geneviève Huisse, Marie-Charlotte Bourrienne, Sawsaneh Alkhaier, Bruno Cassinat, Yacine Boulaftali, Jérôme Debus, Martine Jandrot-Perrus, Christine Chomienne, Christine Dosquet and Nadine Ajzenberg

Table S1. Biological characteristics of ET and PV patients.

Parameters	ET (*n*=35)	PV (*n*=50)	*p*-Value
Platelets, 109/L	439 (323-645)	252 (169-334)	<0.0001
Hemoglobin, g/dl	13.6 (12.8-14.8)	14.5 (14.0-15.7)	0.01
Hematocrit, %	40.7 (37.8-44.1)	44.2 (41.2-47.1)	0.001
Leukocytes, 109/L	6.3 (5.1-8.1)	5.4 (4.5-6.8)	0.05
JAK2V617F, n (%)	18 (51)	49 (98)	<0.0001
Mutations			
JAK2V617F	18 (51.5)	49 (98)	<0.0001
CALR	11 (31.5)	0 (0)	
JAK2 exon 12	0 (0)	1 (2)	
MPL	1 (3)	0 (0)	
Triple negative	5 (14)	0 (0)	
vWF antigen, %	156 (100-208)	157 (111-203)	0.7
vWF activity, %	117 (98-168)	146 (108-185)	0.1
SIPA, %	14 (3.4-23)	17.9 (9.9-31.3)	0.05
P-selectin, %	1.1 (0.4-1.5)	1.1 (0.4-2.0)	0.6
Activated GpIIbIIIa, %	0.9 (0.3-3.3)	1.3 (0.3-4.4)	0.5
PMA, %	7.0 (5.7-10.3)	8.8 (6.9-13.7)	0.05
PNA, %	9.4 (7.2-15.3)	8.0 (6.5-11.4)	0.1
VIII:C,%	126 (100-213)	155 (118-180)	0.4
Fibrinogen, g/dL	3.5 (2.9-4.0)	3.4 (3.0-3.8)	0.8
Protein S, %	84 (72-100)	74 (63-91)	0.06
Protein C, %	117 (100-130)	116 (88-128)	0.4
Antithrombin, %	112 (106-120)	106 (99-111)	0.002
TG without thrombomodulin			
Peak, nM	199 (142-271)	181 (125-234)	0.5
ETP, nM.min	1161 (1021-1415)	1165 (907-1361)	0.7
Velocity, nM/min	58 (32-90)	48 (35-72)	0.4
TG with thrombomodulin			
Peak, nM	86 (39-142)	86 (47-193)	0.4
ETP, nM.min	415 (178-581)	394 (222-810)	0.5
Velocity, nM/min	35 (13-60)	43 (17-94)	0.2

ET: Essential Thrombocytemia, ETP: endogenous thrombin potential, PMA: platelet-monocyte aggregates, PNA: platelet-neutrophil aggregates, PV : Polycythemia Vera, SIPA: shear-induced platelet aggregation, TG: thrombin generation. Results are presented as number (percentage) for categorical variables or as median (IQR) for continuous variables; p-value for Chi-square or Mann-Whitney test.

Table S2. Biological resistance to aspirin according to treatment group.

**Status**	**NT (*n*=18)**	**HU (*n*=30)**	**IFN (*n*=25)**	***p*-Value**
Sensitive	14 (77)	22 (73)	22 (88)	0.4
Resistant	4 (33)	8 (27)	3 (12)	

HU: Hydroxyurea, IFN: Interferon-treated patients, NT: not treated with a cytoreductive drug at the inclusion. Results are presented as number (percentage); p-value for Chi-square.

© 2020 by the authors. Licensee MDPI, Basel, Switzerland. This article is an open access article distributed under the terms and conditions of the Creative Commons Attribution (CC BY) license (http://creativecommons.org/licenses/by/4.0/).
